# Turning the ‘Mustard Oil Bomb’ into a ‘Cyanide Bomb’: Aromatic Glucosinolate Metabolism in a Specialist Insect Herbivore

**DOI:** 10.1371/journal.pone.0035545

**Published:** 2012-04-20

**Authors:** Einar J. Stauber, Petrissa Kuczka, Maike van Ohlen, Birgit Vogt, Tim Janowitz, Markus Piotrowski, Till Beuerle, Ute Wittstock

**Affiliations:** 1 Institute of Pharmaceutical Biology, Technische Universität Braunschweig, Braunschweig, Germany; 2 Department of Plant Physiology, Ruhr-Universität Bochum, Bochum, Germany; French National Centre for Scientific Research, Université Paris-Sud, France

## Abstract

Plants have evolved a variety of mechanisms for dealing with insect herbivory among which chemical defense through secondary metabolites plays a prominent role. Physiological, behavioural and sensorical adaptations to these chemicals provide herbivores with selective advantages allowing them to diversify within the newly occupied ecological niche. In turn, this may influence the evolution of plant metabolism giving rise to e.g. new chemical defenses. The association of Pierid butterflies and plants of the Brassicales has been cited as an illustrative example of this adaptive process known as ‘coevolutionary armsrace’. All plants of the Brassicales are defended by the glucosinolate-myrosinase system to which larvae of cabbage white butterflies and related species are biochemically adapted through a gut nitrile-specifier protein. Here, we provide evidence by metabolite profiling and enzyme assays that metabolism of benzylglucosinolate in *Pieris rapae* results in release of equimolar amounts of cyanide, a potent inhibitor of cellular respiration. We further demonstrate that *P. rapae* larvae develop on transgenic Arabidopsis plants with ectopic production of the cyanogenic glucoside dhurrin without ill effects. Metabolite analyses and fumigation experiments indicate that cyanide is detoxified by β-cyanoalanine synthase and rhodanese in the larvae. Based on these results as well as on the facts that benzylglucosinolate was one of the predominant glucosinolates in ancient Brassicales and that ancient Brassicales lack nitrilases involved in alternative pathways, we propose that the ability of Pierid species to safely handle cyanide contributed to the primary host shift from Fabales to Brassicales that occured about 75 million years ago and was followed by Pierid species diversification.

## Introduction

**Figure 1:**
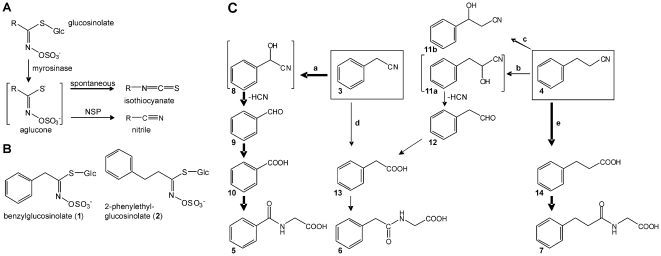
The glucosinolate-myrosinase system and proposed pathways of aromatic nitrile metabolism in *P. rapae* larvae. **A.** Myrosinase-catalyzed hydrolysis of glucosinolates upon plant tissue disruption yields an unstable aglucone which most commonly rearranges to a toxic isothiocyanate. Larvae of *P. rapae* redirect glucosinolate breakdown to the formation of simple nitriles by the gut nitrile-specifier protein (NSP). R, variable side chain. **B.** Examples of glucosinolates with aromatic (i.e. benzene ring-containing) side chains. **C.** Upon ingestion of plant material by *P. rapae* larvae, **1** and **2** are converted to phenylacetonitrile (**3**) and 3-phenylpropionitrile (**4**), respectively. These undergo further metabolism to the glycine conjugates **5–7** which are excreted with the feces. The major metabolite of **1** is hippuric acid (*N*-benzoylglycine, **5**; 23, 24), the major metabolite of **2** is *N*-(3-phenylpropionyl)glycine (**7**, this study). *N*-phenylacetylglycine (**6**) is formed as a minor metabolite from both glucosinolates. This study establishes the pathways from **3** and **4** to **5–7**. While the conversion of **3** to **5** involves a C1-loss through HCN release (route **a**), the side chain of **4** is maintained throughout its major metabolic pathway (route **e**). Reactions **a**, **b** and **c** are catalyzed by an NADPH-dependent microsomal enzyme activity. Reaction **d**, and likely, reaction **e** involve nitrilase activity from the ingested plant material. Compounds **9**, **11b** and **12** were detected as intermediates in this study. Bold and thin arrows indicate major and minor metabolic pathways, respectively.

Insects that feed on plants are confronted with some major challenges. Not only are many plant organs low in protein and equipped with physical barriers such as trichomes and waxes, but they are also protected against herbivory by an array of defensive chemicals derived from secondary metabolism [1], [2]. Chemical defenses may act as toxins, deterrents or repellents or may indirectly affect insect growth and development [1], [2]. While the diversity of plant secondary metabolites is thought to be shaped, among others, by selection pressures exerted by herbivores [3], evolution of plant chemistry may, in turn, affect the evolution of herbivores in an ‘evolutionary armsrace’ [4]–[6]. This means that herbivores develop behavioral and/or biochemical adaptations in response to the chemistry of potential food plants and even become specialized on plants that produce a certain group of secondary metabolites. This is often accompanied by sensory adaptations that allow the insect to positively select a suitable host plant based on the presence of the chemical that it has adapted to. The glucosinolate-myrosinase system or ‘mustard oil bomb’ [7] is one of the best studied plant chemical defenses. Glucosinolates are amino-acid derived thioglucosides (Fig. 1A, B) that are present in essentially all genera of the Brassicales [8]–[10]. Glucosinolate biosynthesis has long been thought to have evolved from a ‘cyanogenic predisposition’ about 85–90 million years ago [11]–[13]. This means that mutations in genes involved in the biosynthesis of cyanogenic glucosides, a group of amino-acid derived β-glucosides of α-hydroxynitriles that are widely distributed in the plant kingdom, led to changed enzyme activities yielding new biosynthetic intermediates that were finally metabolized into glucosinolates, a trait that is restricted to the Brassicales and the genus Drypetes (Putranjivaceae) [14]. Recent data suggest independent evolution of glucosinolates and cyanogenic glucosides as metabolites of reactive oximes formed by ancestral cytochrome P450 enzymes [15].

In contrast to many other chemical defenses, the glucosinolates themselves are non-toxic. They become activated upon tissue damage when endogenous thioglucosidases, the myrosinases, are released from their separate storage compartments and hydrolyze the glucosinolates to biologically active products that play a role in plant-pathogen and plant-insect interactions [9], [16] (Fig. 1A). The most intensely studied hydrolysis products, the isothiocyanates (mustard oils), are very reactive and have been shown to be toxic to bacteria, fungi, nematodes and insects and have attracted a lot of interest as anticarcinogenic compounds in our diet [17], [18].

The association of Pierid butterflies with glucosinolate-containing plants began only about 10 million years after the evolution of the glucosinolate-myrosinase system in plants [6]. The key evolutionary innovation that is thought to have allowed colonization of glucosinolate-containing plants by Pierid butterflies has been identified as the gut nitrile-specifier protein (NSP) that enables Pierid larvae to circumvent the harmful effects of the glucosinolate-myrosinase system [6]. Larval NSP redirects plant myrosinase-catalyzed glucosinolate hydrolysis to nitriles instead of the toxic isothiocyanates [19] (Fig. 1A). While aliphatic nitriles are excreted unchanged with the feces, nitriles derived from aromatic glucosinolates undergo further metabolism [20]–[23] (Fig. 1C). In *Pieris rapae*, one of the most widespread butterflies of the northern hemisphere and a major agricultural pest, the nitrile derived from benzylglucosinolate, phenylacetonitrile, is metabolized to hippuric acid and minor amounts of *N*-phenylacetylglycine and *N*-benzoylisoserine [20], [21] (Fig. 1C). Isotopic labeling experiments suggested that the formation of *N*-phenylacetylglycine likely proceeds through the conversion of phenylacetonitrile into phenylacetic acid, presumably catalyzed by a nitrilase, followed by conjugation with glycine [20]. However, formation of hippuric acid from phenylacetonitrile involves the loss of one carbon atom and can not be easily explained by a nitrilase reaction.

Here, we provide evidence that the C1 loss during aromatic nitrile metabolism proceeds through an α-hydroxylation of the nitrile yielding an unstable α-hydroxynitrile that decomposes spontaneously to an aldehyde and cyanide turning the ‘mustard oil bomb’ into a ‘cyanide bomb’ inside the larvae. This result motivated us to test the ability of *P. rapae* larvae to develop on transgenic Arabidopsis plants with ectopic production of a cyanogenic glucoside [24]. We found that the larvae were able to tolerate high levels of the cyanogenic glucoside dhurrin without ill effects. Further experiments suggest that they are able to efficiently detoxify cyanide by the activities of β-cyanoalanine synthase and rhodanese. This ability might have contributed to the primary host shift from Fabales to glucosinolate-containing plants which led to species diversification within the Pierinae [25].

## Results

### The major routes of metabolism of benzylglucosinolate and phenylethylglucosinolate in *P. rapae* larvae are different

To compare the metabolism of benzylglucosinolate and 2-phenylethylglucosinolate, *P. rapae* larvae were fed *A. thaliana* Col-0 leaves to which either of the two exogenous glucosinolates had been applied. Aqueous feces extracts of the larvae were then analyzed by HPLC-MS. Feces from larvae that had ingested phenylethylglucosinolate contained *N*-benzoylglycine, *N*-phenylacetylglycine, and *N*-(3-phenylpropionyl)glycine (Fig. S1). Ingestion of benzylglucosinolate led to the formation of *N*-benzoylglycine and *N*-phenylacetylglycine (Fig. S1) as reported previously [20], [21]. Background levels of *N*-benzoylglycine and *N*-phenylacetylglycine, but not of *N*-(3-phenylpropionyl)glycine, were found in feces extracts from larvae fed Col-0 leaves to which no glucosinolate had been applied.

**Figure 2:**
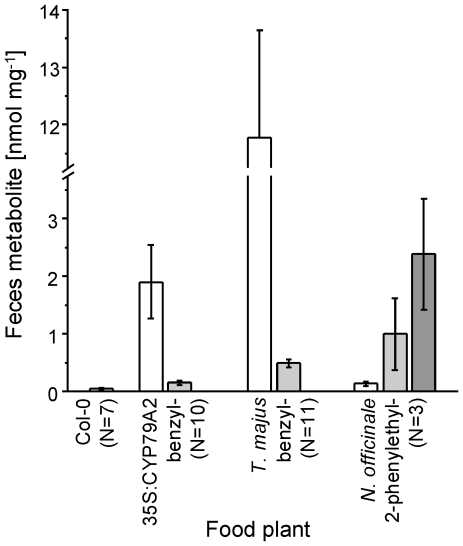
Differential metabolism of aromatic glucosinolates with different side chain lengths in *P. rapae* larvae. Feces were collected from *P. rapae* larvae that had fed on leaves of *A. thaliana* Col-0, 35S:CYP79A2, *Tropaeolum majus*, or *Nasturtium officinale*. Glycine conjugates **5** (white), **6** (light-gray), and **7** (dark-gray) were quantified in feces extracts by HPLC-MS using ^13^C-labeled **5**, **6**, and **7** as standards. Means ± SD are given with N (number of biological replicates). Each replicate represents a pair of larvae.

For a quantitative comparison of metabolite profiles, leaves of either *Nasturtium officinale* (which produce primarily phenylethylglucosinolate [26]), *A. thaliana* 35S:CYP79A2 or *Tropaeolum majus* (both of which produce high amounts of benzylglucosinolate [27], [28]), or Col-0 plants which do not produce aromatic glucosinolates in leaves [29], were fed to *P. rapae* larvae. In feces extracts of larvae that had ingested *N. officinale* leaves, *N*-(3-phenylpropionyl)glycine was the most abundant conjugate followed by *N*-phenylacetylglycine and *N*-benzoylglycine (Fig. 2). Metabolism of benzylglucosinolate from either 35S:CYP79A2 or *T. majus* resulted in formation of *N*-benzoylglycine as the major metabolite and small amounts of *N*-phenylacetylglycine (Fig. 2). Thus, the C3 chain of 2-phenylethylglucosinolate is maintained throughout the major metabolic pathway of this glucosinolate in *P. rapae* larvae. However, a considerable amount of ingested 2-phenylethylglucosinolate also undergoes a C1 loss. In contrast, the major route of benzylglucosinolate metabolism includes a C1 loss from the C2 chain of this glucosinolate.

### Plant nitrilases are involved in metabolism of aromatic glucosinolates without carbon loss

As aliphatic as well as aromatic glucosinolate metabolism in *P. rapae* is known to proceed through the corresponding nitrile intermediates due to the action of plant myrosinase in conjunction with larval NSP [19], we tested if plant nitrilases are involved in aromatic nitrile metabolism in the larvae using an *A. thaliana Nit2*-RNAi mutant. This mutant is devoid of any nitrilase activity detectable with the substrate 3-phenylpropionitrile (Fig. S2). To allow the use of intact plants for feeding experiments, we crossed benzylglucosinolate-producing *A. thaliana* 35S:CYP79A2 plants with the *Nit2*-RNAi mutant. The 35S:CYP79A2×*Nit2*-RNAi plants (F1) had no detectable nitrilase activity, but contained benzylglucosinolate (Fig. S2). For metabolite analysis, we collected feces from larvae that had fed either on Col-0, 35S:CYP79A2 or 35S:CYP79A2×*Nit2*-RNAi plants and analyzed aqueous feces extracts by HPLC-MS (Table 1). The percentage of *N*-phenylacetylglycine of the total amount of glycine conjugates was significantly lower in feces from larvae fed 35S:CYP79A2×*Nit2*-RNAi plants than in feces from larvae fed 35S:CYP79A2 plants and equal to the background levels found in feces from larvae fed Col-0 plants. In contrast, there was no difference in the levels of the major metabolite *N*-benzoylglycine in the three extracts. This suggests that the formation of *N*-phenylacetylglycine, but not the formation of *N*-benzoylglycine associated with a C1 loss, depends on plant nitrilase activity.

**Table 1:** Plant nitrilase activity contributes only to the minor pathway of benzylglucosinolate metabolism in *P. rapae* larvae.

Genotype of food plant	N-benzoyl-glycine 5 (%)	N-phenyl-acetylglycine 6 (%)	N
35S:CYP79A2	92.2±33.5	7.6±1.8 *	10
35S:CYP79A2×Nit2-RNAi	96.4±18.0	2.7±1.2 *	6
Col-0	96.2±26.9	1.6±0.3	7

Feces were collected from *P. rapae* larvae that had fed on leaves of *A. thaliana* of the given genotypes. Glycine conjugates **5** and **6** were quantified in feces extracts by HPLC-MS using ^13^C-labeled standards. Means ± SD are given with N as the number of biological replicates. Each replicate represents a pair of larvae. The asterisk indicates a significant difference (Mann-Whitney U-test, P = 0.001).

### The C1 loss proceeds through an α-hydroxynitrile that decomposes to an aldehyde and HCN

**Figure 3:**
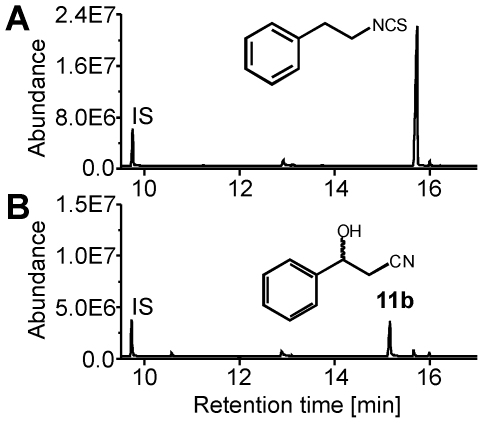
Organic phase metabolites of 2-phenylethylglucosinolate in plant homogenates and *P. rapae* larvae. Dichloromethane extracts of *N. officinale* leaf autolysates (**A**) and the organic phase of dichloromethane/water extracts of feces from *P. rapae* larvae that had fed on *N. officinale* leaves (**B**) were analyzed by GC-MS. Shown are total ion current traces. IS, internal standard.

When the dichloromethane phase of feces extracts from *N. officinale* fed larvae (see above) was subjected to GC-MS, we identified 3-hydroxy-3-phenylpropionitrile (the stable β-hydroxynitrile of 3-phenylpropionitrile; Fig. 3). In contrast, autolysates of *N. officinale* leaves contained 2-phenylethylisothiocyanate, the main hydrolysis product of 2-phenylethylglucosinolate, but no 3-hydroxy-3-phenylpropionitrile. This led us to propose that a hydroxylation may also happen at the α-position yielding the unstable α-hydroxynitrile that would spontaneously decompose into phenylacetaldehyde and cyanide, explaining the C1 loss. In fact, phenylacetaldehyde was present in the dichloromethane phase in minor amounts. This result suggested that the benzylglucosinolate-derived phenylacetonitrile may yield benzaldehyde and cyanide after α-hydroxylation and decomposition.

**Figure 4:**
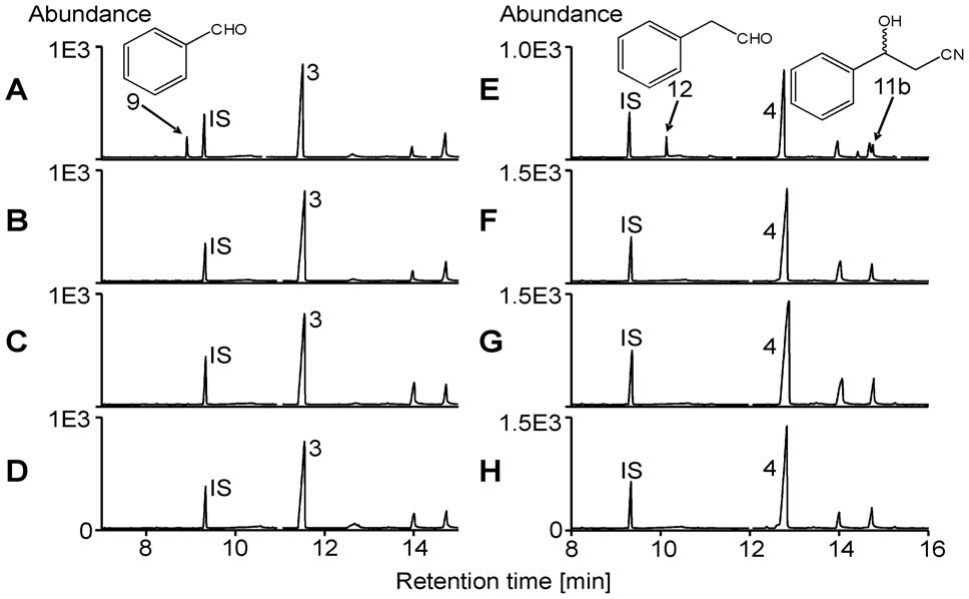
NADPH-dependent hydroxylation of aromatic nitriles by *P. rapae* gut microsomes. Larval gut microsomes were incubated with 2.5 mM phenylacetonitrile **3** (**A**–**D**) or 2.5 mM 3-phenylpropionitrile **4** (**E**–**H**) for 45 min at 31°C in the presence (**A**, **C**–**E**, **G**, **H**) or absence (**B**, **F**) of NADPH. In **C** and **G**, microsomes were flushed with CO prior to addition of NADPH. In **D** and **H**, microsomes were heated (95°C, 5 min) prior to the assay. Assays were extracted with dichloromethane, and the organic phases analyzed by GC-MS. Shown are total ion current traces. IS, internal standard.

**Figure 5:**
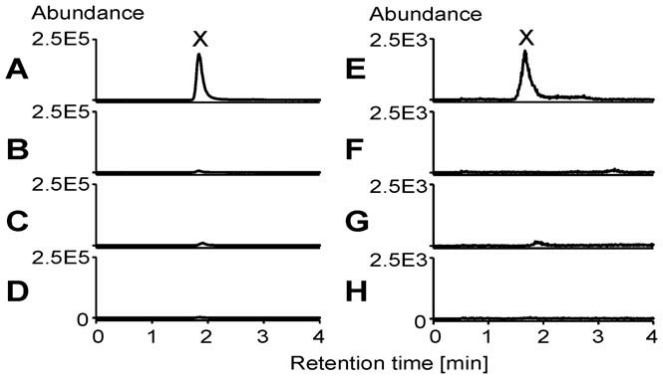
Cyanide is released as a consequence of aromatic nitrile hydroxylation by *P. rapae* gut microsomes. Larval gut microsomes were incubated with 2.5 mM phenylacetonitrile **3** (**A**–**D**) or 2.5 mM 3-phenylpropionitrile **4** (**E**–**H**) in the presence (**A**, **C**–**E**, **G**, **H**) or absence (**B**, **F**) of NADPH. Cyanide was captured by derivatization. Shown are HPLC-MS/MRM traces of the derivatization product (X). In **C** and **G**, microsomes were flushed with CO prior to addition of NADPH. In **D** and **H**, microsomes were heated (95°C, 5 min) prior to the assay.

In order to test our hypothesis, we performed enzyme assays using larval gut extracts and phenylacetonitrile or 3-phenylpropionitrile as substrates in the presence and absence of exogenously applied NADPH (Fig. 4). We detected the aldehydes of both substrates, presumably formed as decomposition products of the α-hydroxynitriles, as well as the β-hydroxynitrile of 3-phenylpropionitrile in assay mixtures containing the microsomal protein fraction of gut extracts and NADPH (Figs. 4A, 4E), but not in assay mixtures containing the soluble protein fraction or lacking NADPH (Figs. 4B, 4F, soluble fraction not shown). Bubbling CO through the microsome preparation before addition of NADPH led to loss of activity (Figs. 4C, 4G). This suggests that the C1 loss during aromatic nitrile metabolism in *P. rapae* larvae likely proceeds through a cytochrome P450 monooxygenase (cytP450)-catalyzed α-hydroxylation followed by decomposition to an aldehyde and cyanide thus turning the ‘mustard oil bomb’ into a ‘cyanide bomb’. To further substantiate this finding, we conducted the same set of microsomal enzyme assays as above, but capturing cyanide by derivatization [30]. Only in those assay mixtures that were able to hydroxylate our substrates, were we able to identify the product of cyanide derivatization (Fig. 5). Together with the results presented in Fig. 4, this confirms that microsomal hydroxylation of phenylacetonitrile or 3-phenylpropionitrile yields α-hydroxynitriles which decompose to cyanide and the corresponding aldehydes.

### *P. rapae* larvae tolerate high levels of cyanide in their diet

**Figure 6:**
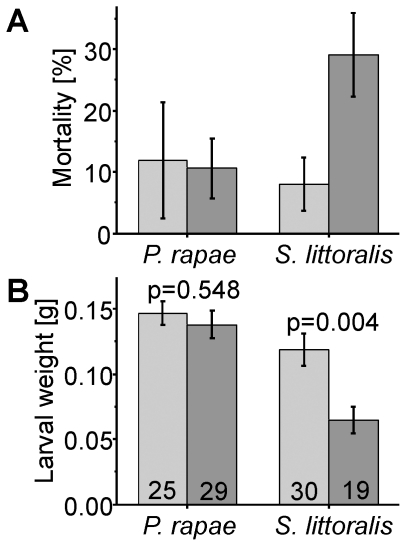
Performance of *P. rapae* larvae on cyanogenic plants. *P. rapae* and *S. littoralis* larvae were allowed to feed on either *A. thaliana* Col-0 wildtype (gray) or cyanogenic *A. thaliana* 3x/dhurrin plants (dark grey). After 10 d, surviving larvae were counted and weighted. Larval mortality is given as means ± SEM of three independent experiments. Larval weights are given as means ± SEM from one out of three independent experiments. Results of all experiments are shown in Table S1. Numbers in the bars indicate N (number of surviving individuals).

When *P. rapae* larvae feed on *T. majus* plants, they ingest high levels of benzylglucosinolate. As shown above, the major pathway of benzylglucosinolate metabolism is associated with the release of equimolar amounts of cyanide. Consumption of 1 cm^2^ of a leaf of *T. majus* (approximately 50 mg with 5 µmol/g benzylglucosinolate) by an L5 larva would result in the release of about 7 µg cyanide in the insect gut within a period of less than 1 h. This would correspond to 50 mg cyanide per kg body weight. For comparison, LD_50_ values for humans are at about 1–2 mg/kg upon peroral administration. Thus *P. rapae* larvae seem to be exceptionally tolerant to cyanide. To test cyanide tolerance in *P. rapae*, we performed bioassays in which we fed leaves of transgenic *A. thaliana* plants (3x/dhurrin) engineered to produce high levels of the cyanogenic glucoside dhurrin (1–4 mg/g fresh weight [24]) to larvae of *P. rapae* and, for comparison, to the generalist lepidopteran herbivore *Spodoptera littoralis* that is known to be extremely polyphagous and resistant to many insecticides. *P. rapae* larvae completed development on the cyanogenic plants and were not affected in survival rate or growth as compared to larvae raised on *A. thaliana* Col-0 wildtype plants (Fig. 6, Table S1). In one experiment, they even grew significantly faster on cyanogenic than on wildtype plants. In contrast, average survival of *S. littoralis* larvae was reduced by more than 20% on cyanogenic plants as compared to wildtype plants, and the surviving larvae grew significantly slower on the cyanogenic than on wildtype plants (Fig. 6, Table S1).

### *P. rapae* larvae incorporate cyanide into β-cyanoalanine and SCN^−^

**Figure 7:**
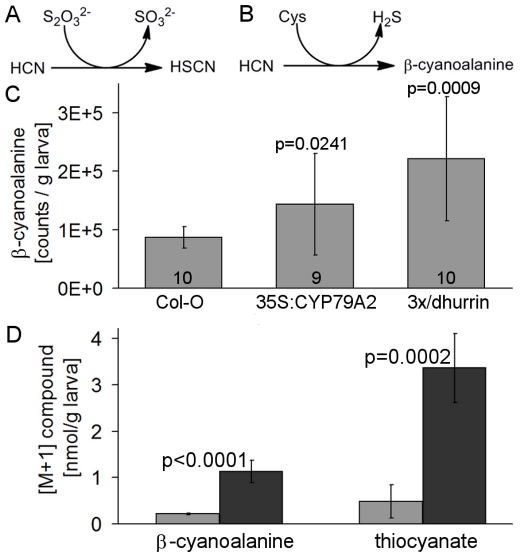
Detoxification of cyanide in *P. rapae*. **A.** Scheme of the reaction catalyzed by rhodanese. **B.** Scheme of the reaction catalyzed by β-cyanoalanine synthase. **C.** β-Cyanoalanine content in *P. rapae* larvae after nine days of feeding on wildtype, benzylglucosinolate-rich (35S:CYP79A2) and cyanogenic (3x/dhurrin) *A. thaliana* plants. Larvae were extracted with dichloromethane and water. The aqueous phase was analyzed by HPLC-MS. Data are means ± SD. N (number of larvae analyzed) is given in the bars, p values (t-test) for the comparison with Col-0 above the bars. **D.** Quantitative analysis of β-cyanoalanine and SCN^−^ with M+1 after 24 h [^15^N]HCN fumigation of the larvae. Each bar represents the mean ± SD of N = 16 individual larvae. P values (t-test) are given above the bars for the comparison of fumigated (dark-grey bars) to non-fumigated larvae (light-grey bars). Data in **C** and **D** are each from one out of at least three independent experiments that all showed significant differences (p<0.05).

To identify possible pathways of cyanide detoxification in *P. rapae* (Fig. 7A, B), we first quantified the levels of β-cyanoalanine in larvae after consumption of benzylglucosinolate or dhurrin-producing transgenic *A. thaliana* plants. Extracts of larvae that had fed on these plants contained significantly more β-cyanoalanine than those obtained from larvae that had fed on wildtype plants devoid of both compounds (Fig. 7C). Next, we followed the incorporation of cyanide into β-cyanoalanine and SCN^−^ in fumigation experiments in which larvae were kept in a [^15^N]HCN atmosphere before analysis for [M+1]β-cyanoalanine and [M+1]SCN^−^ which result from the incorporation of ^15^N and naturally occuring ^13^C. [^15^N]HCN-fumigated larvae contained about ten times more [M+1]β-cyanoalanine and [M+1]SCN^−^ than non-fumigated larvae confirming detoxification of cyanide by formation of β-cyanoalanine and SCN^−^ (Fig. 7D).

## Discussion

As a strong inhibitor of cellular respiration, cyanide is universally toxic and has been shown to be an effective defense against herbivory [31], [32]. Previous research has identified larval NSP as a key evolutionary innovation that allowed Pierid butterflies to colonize plants defended by the glucosinolate-myrosinase system [6]. Our present study shows that the adaptive significance of NSP was dependent on the ability of Pierid larvae to detoxify cyanide. Based on the phylogeny of the Brassicales and reports on the distribution of glucosinolates, aromatic glucosinolates like those derived from phenylalanine, are the most widespread and often predominant glucosinolates within basal families of the Brassicales such as the Tropaeolaceae and Caricaceae [8], [10], [33]. As the host shift of Pierids from Fabales to Brassicales is thought to have happened only about 10 million years after the appearance of the Brassicales [6], ancestral Brassicales-feeding Pierid species would have been confronted with phenylalanine-derived glucosinolates. Using metabolite profiling (Fig. S1, Figs. 2, 3) and microsomal enzyme assays (Figs. 4, 5), we provide evidence that benzylglucosinolate metabolism in *P. rapae* is linked to cyanide production. Thus, the ancestral Pierids must have been able to detoxify high levels of cyanide as a prerequisite for colonization of glucosinolate-containing plants. We demonstrate the exceptional ability of *P. rapae* to tolerate high cyanide levels by feeding experiments with transgenic *A. thaliana* that accumulate the cyanogenic glucoside dhurrin. While the generalist lepidopteran *S. littoralis* (Fig. 6) as well as the specialist coleopteran *Phyllotreta nemorum* (Chrysomelidae) [24] are strongly affected in their survival and growth when raised on cyanogenic plants as compared to *A. thaliana* wildtype, *P. rapae* develops without ill effects on cyanogenic plants (Fig. 6). As species of the Fabales order were among the major food plants of Pierids prior to the host shift to Brassicales [34], and since cyanogenic glucoside-producing species are common in the Fabales [35], the ability to detoxify cyanide may have evolved in response to the presence of cyanogenic glucosides in the Fabales food plants. Alternatively, it is possible that the Pieridae or their ancestors might have been cyanogenic themselves in ancient times. Indeed, Pieridae are relatively closely related to the Zygaenidae which have been demonstrated to be able to synthesize and safely metabolize cyanogenic compounds [36], [37]. While most species likely lost the ability to handle larger amounts of cyanide, *P. rapae* might be one of those species in which this ability was maintained as it provided some kind of selective advantage, most likely in conjunction with the preferred food plants.

Two enzymes, rhodanese and β-cyanoalanine synthase, are known to function in cyanide detoxification in insects [38]–[40], but have primarily been investigated in mammals, plants, and microorganisms [41], [42]. The finding that a diet containing benzylglucosinolate or the cyanogenic glucoside dhurrin increases β-cyanoalanine levels in *P. rapae* together with the formation of [^15^N]β-cyanoalanine and [^15^N]SCN^−^ after fumigation of larvae with [^15^N]HCN (Fig. 7) provides evidence for a role of these pathways in cyanide detoxification in *P. rapae*. In support of this result, β-cyanoalanine synthase and rhodanese activities were detected in gut tissue of *P. rapae* larvae (data not shown). In plants, β-cyanoalanine is converted to aspartic acid and asparagine by nitrilase NIT4 homologs [43]. It is currently not known if this conversion takes also place in insects, but if it did, it would allow the larvae to channel the glucosinolate-derived cyanide into amino acid metabolism. Thus, instead of acting as a defense, benzylglucosinolate may provide *P. rapae* with valuable nutrients. First, one molecule of glucose and one molecule of sulfate are released per molecule of benzylglucosinolate ingested and secondly, trapping of the glucosinolate-derived cyanide as β-cyanoalanine would likely equal out the investment of glycine used to form hippuric acid for excretion of the remainder of the glucosinolate skeleton thus avoiding a net loss of nutrients during detoxification.

Previous research has suggested that ingested plant nitrilases are involved in phenolic glucosinolate metabolism in *P. rapae* [22], [23]. In support of this, we found plant nitrilase activity to contribute to the formation of *N*-phenylacetylglycine after larvae had ingested benzylglucosinolate-containing plant material (Tab. 1). However, we also demonstrated that formation of *N*-phenylacetylglycine (pathway **d**, Fig. 1C) is only a minor pathway of benzylglucosinolate metabolism in *P. rapae* (Fig. 2). In contrast, formation of *N*-(3-phenylpropionyl)glycine is a major pathway (pathway **e**, Fig. 1C) when *P. rapae* larvae metabolize 2-phenylethylglucosinolate (Fig. 3), a representative of chain-elongated phenylalanine-derived glucosinolates which do not occur in basal Brassicales families [10]. Based on the observations that plant nitrilases may be active in the insect gut (see above), that *A. thaliana* nitrilases NIT1-NIT3 prefer 3-phenylpropionitrile over phenylacetonitrile [44] and that nitrilases of other species also seem to be substrate-specific [45], the predominance of one of the pathways may depend on the substrate specificity of the nitrilase present in the ingested plant material. However, nitrilases of the NIT1 family (those that have been reported to accept glucosinolate-derived nitriles) have only been reported in the Brassicaceae, one of the youngest families of the Brassicales, and seem to be absent from other families [46]. This is in agreement with our finding that hippuric acid is the major metabolite of benzylglucosinolate when larvae ingest leaves of *T. majus* (Fig. 2). Taken together, this concurs with the requirement for an efficient detoxification system for cyanide in early glucosinolate-feeding Pierid species.

The involvement of two different groups of plant chemical defenses in speciation of one group of herbivores has rarely been studied. Our data link cyanogenic glucoside and glucosinolate metabolism in *P. rapae* providing new insights into the biochemical bases of adaptations of insect herbivores to the complex chemistry of their host plants in a coevolutionary context. The detoxification of benzylglucosinolate in *P. rapae* further highlights the biochemical similarities of the metabolism of cyanogenic glucosides and glucosinolates.

## Materials and Methods

### Plants

*A. thaliana*, *N. officinale*, and *T. majus* plants were grown in a controlled environment chamber at 22°C and 55% humidity with a photoperiod of 10 h, at a light intensity of 230 µmol m^−2^ s^−1^. *A. thaliana* genotypes were: wild-type Columbia-0 (Col-0), transgenic 35S:CYP79A2 (high levels of benzylglucosinolate [27]), transgenic *Nit2*-RNAi (see below), 35S:CYP79A2×*Nit2*-RNAi (F1), transgenics with overexpression of CYP79A1, CYP71E1, and sbHMNGT termed 3x/dhurrin in the present paper and kindly provided by Søren Bak, Copenhagen University (high levels of the cyanogenic glucoside dhurrin [24]). All transgenic plants were in the Col-0 background.

### Generation of the Nit2-RNA_i_ plants

The exon 2/intron 2 section and exon 2 of the *NIT2* gene from *A. thaliana* were amplified from genomic DNA using the primers AtNIT2E2I2_Forw 5′-TAT TCT AGA AAA AGG CGA ACA AGT TTA TTG TGG, AtNIT2E2I2_Rev 5′-TAT GAA TTC CTA CAG GTT CAT CAC ACA AAA AAA G, AtNIT2E2_Forw 5′-TAT GAG CTC AAA AGG CGA ACA AGT TTA TTG TGG AG and AtNIT2E2_Rev 5′-TAT GAA TTC CAG GAA CTT TAA TAG CAG AAG CAT G. PCR products were cloned into the pGEM-T vector (Promega) and verified by sequencing. Exon 2 was cloned in reverse complement behind the exon 2/intron 2 fragment using EcoRI. The whole construct was then cloned into pCBi18 [47] behind the CaMV35S promoter using XbaI and SacI, thus replacing the *uidA* gene in pCBi18. *A. thaliana* plants were transformed with *Agrobacterium tumefaciens* GV3101 using the floral-dip method [48]. The selected homozygous T2 line carries one T-DNA integration, is devoid of NIT1 and NIT2 protein as judged by western blotting, resistant against exogenously applied indole-3-acetonitrile and displays no nitrilase activity with 3-phenylpropionitrile as substrate.

### Insect rearing

A culture of *P. rapae* butterflies was kept on Brussels sprouts (*Brassica oleracea* ssp. *oleracea*, cv. Rosella) plants in a controlled environment chamber at 25°C and 60% relative humidity with a photoperiod of 16 h. The culture originated from individuals donated by J. van Loon (Wageningen University, Wageningen, The Netherlands) in January 2007 and was supplemented by individuals collected in the Medicinal Plant Garden of the Institute of Pharmaceutical Biology at the Technische Universität Braunschweig, Germany (no collection permission required for institute members). *P. rapae* is not an endangered or protected species in Europe. For experiments with *T. majus*, larvae were either hatched on these plants or transferred to them immediately after hatching. *S. littoralis* were hatched from eggs that originated from Syngenta Crop Protection AG (Stein, Switzerland) and were kindly provided by K. Schramm (Max Planck Institute for Chemical Ecology, Jena).

### Chemicals

Benzylglucosinolate was purified from *Lepidium sativum* seeds and 2-phenylethylglucosinolate was isolated from *N. officinale* seeds as described in [49]. β-Cyanoalanine and [^13^C_2_]-Glycine were purchased from Sigma-Aldrich. Isotopically labeled *N*-benzoyl-[^13^C_2_]-glycine, *N*-phenylacetyl-[^13^C_2_]-glycine, and *N*-(3-phenylpropionyl)-[^13^C_2_]-glycine were synthesized as described in [50], re-crystallized from ethylacetate and their identities were confirmed by ^1^H-NMR and EI-MS (see below).

### Confirmation of chemically synthesized compounds by ^1^H-NMR and EI-MS

EI-MS data were recorded with an MAT 90 mass spectrometer (Thermo Finnigan, Bremen, Germany) and direct insertion probe at 70 eV. ^1^H-NMR spectra were recorded on a Bruker AC 250 (400 MHz) spectrometer using tetramethylsilane as an internal standard. *N*-Benzoyl-[^13^C_2_]-glycine: ^1^H-NMR (400 MHz, CD_3_OD) δ (ppm): 4.12 (dd, 2H, ^1^ *J*_CH_ = 139.3 Hz, ^2^ *J*_CH_ = 5.9 Hz, *CH_2_*), 7.46 (m, 2H, Ar-*H*), 7.57 (m, 1H, Ar-*H*), 7.88 (m, 2H, Ar-*H*); EI-MS *m/z* (rel. abund. (%)): 181 (M^+^, 3), 163 (3), 149 (2), 136 (35), 135 (29), 118 (6), 105 (78), 92 (26), 91 (32), 77 (100), 65 (7), 51 (31). *N*-phenylacetyl-[^13^C_2_]-glycine: ^1^H-NMR (400 MHz, CD_3_OD) δ (ppm): 3.61 (s, 2H, Ar-*CH_2_*), 3.92 (dd, 2H, ^1^ *J*_CH_ = 139.2 Hz, ^2^ *J*_CH_ = 5.9 Hz, *CH_2_*), 7.26 (m, 1H, Ar-*H*), 7.32 (m, 2H, Ar-*H*), 7.34 (m, 2H, Ar-*H*); EI-MS *m/z* (rel. abund. (%)): 195 (M^+^, 13), 150 (8), 118 (20), 104 (3), 92 (100), 91 (95), 75 (19), 65 (21), 57 (12). *N*-(3-phenylpropionyl)-[^13^C_2_]-glycine. ^1^H-NMR (400 MHz, CD_3_OD) δ (ppm): 2.44 (m, 2H, Ar-CH_2_-*CH_2_*), 2.81 (m, 2H, Ar-*CH_2_*).3.78 (dd, 2H, ^1^ *J*_CH_ = 139.1 Hz, ^2^ *J*_CH_ = 5.9 Hz, *CH_2_*), 7.08–7.18 (m, 5H, Ar-*H*). EI-MS *m/z* (rel. abund. %): 209 (M^+^, 35), 191 (4), 164 (2), 133 (6), 131 (8), 105 (35), 104 (100), 91 (45), 78 (30), 65 (7), 51 (9).

### GC-MS analysis

GC-MS analysis was done according to [51]. Compounds were identified by comparison of their mass spectra with those of standard substances with the exception of 3-hydroxy-3-phenylpropionitrile which was identified with the NIST database. For analysis of glucosinolate hydrolysis products in leaf homogenates, samples were prepared as described in [51].

### HPLC-MS analysis

Aqueous samples were analyzed using an HP1200 series HPLC instrument (Agilent Technologies, Waldbronn, Germany) equipped with a Hyperclone ODS(C18) column (150×2.0 mm, 5 µM particle size; Phenomenex, Aschaffenburg, Germany) and coupled to a 3200 QTRAP mass spectrometer (ABSciex). Chromatographic and mass-spectrometric conditions were as follows:

Analysis of glycine conjugates **5–7**: Gradient of 0.1% (vol/vol) formic acid in water (solvent A) and 0.1% (vol/vol) formic acid in acetonitrile (solvent B): 6% B (0.5 min), 6–27% B (2.0 min), 27–60% B (6.5 min), 60–99% B (0.5 min), 99% B (1.0 min), 99–5% B (0.5 min), 5% B (5 min), flow rate 0.3 ml/min. Mass spectra were recorded between minutes 4.7 and 9.3, otherwise flow was diverted to waste. The mass spectrometer was operated with a source voltage of 5.5 kV, declustering potential of 25 V, nitrogen for nebulization with the curtain gas, gas 1 and gas 2 settings at 20, 45, and 50, respectively, and a source temperature of 580°C. Nitrogen was used for collision induced dissociation at the medium setting. The collision energy varied between 17 and 27 V. The ion pairs in Multiple Reaction Monitoring (MRM) were: unlabeled/^13^C_2_-labeled *N*-benzoylglycine: 180-105/182-105, unlabeled/^13^C_2_-labeled *N*-phenylacetylglycine: 194-150/196-150, unlabeled/^13^C_2_-labeled *N*-(3-phenylpropionyl)-glycine: 208-105/210-105. Unlabeled metabolites were quantified based on peak areas as compared to those obtained with synthetic ^13^C_2_-standards.

Analysis of the cyanide derivatization product 2-(1-cyano-*2H*-benzo[f]isoindol-2-yl) acetic acid: Gradient of 0.1% (vol/vol) formic acid in water (solvent A) and 0.1% (vol/vol) formic acid in acetonitrile (solvent B): 85% B (0.3 min), 85–65% B (1.7 min), 65–85% B (2.5 min), 85% B (0.5 min), flow rate 0.3 ml/min. Mass spectra were recorded between minutes 0.5 and 4.5, otherwise flow was diverted to waste. The mass spectrometer was operated in the negative mode with a source voltage and declustering potential of −4.5 kV and −15 V, respectively. Gas settings and source temperature were as above. The collision energy was −10 V. The ion pairs monitored to detect ^13^C_2_-labeled or unlabeled 2-(1-cyano-*2H*-benzo[f]isoindol-2-yl) acetic acid were 249-205/251-206.

Analysis of β-cyanoalanine and thiocyanate: Gradient of 0.1% (vol/vol) formic acid in water (solvent A) and 0.1% formic acid in methanol (solvent B): 5% B (2 min), 5–25% B (2 min), 25% B (1 min), 25–95% B (2 min), 95–5% B (0.01 min), 5% B (2 min), flow rate 0.3 ml/min. Mass spectra were recorded starting at 1 min. The mass spectrometer was operated in the negative mode with source voltage and declustering potential of −6 kV and −35 V, respectively, for the determination of β-cyanoalanine and −4.5 kV and −50 V, respectively, for thiocyanate. Gas settings were as above. The source temperature was 630°C. Nitrogen was used for collision induced dissociation at the medium setting. The collision energy was −13 V for β-cyanoalanine and −25 V for thiocyanate. The ion pairs monitored to detect unlabeled and ^15^N-labeled β-cyanoalanine and thiocyanate were 112.7-95.9/113.7-96.9 and 57.9-57.9/58.9-58.9, respectively.

### Feeding experiments with intact plants and external addition of glucosinolates

One ml of 0.2 mM benzylglucosinolate or 2-phenylethylglucosinolate in 50% (vol/vol) ethanol or 1 ml 50% (vol/vol) ethanol was distributed onto the rosette leaves of a six-week-old *A. thaliana* Col-0 plant. After the solvent had evaporated, *P. rapae* larvae were allowed to feed on each of the plants. A wax paper collar was placed under the leaves to collect the feces. For each condition, approximately 150 mg feces were continuously collected and stored on ice. To each sample, 1 ml water was added, samples were extracted with dichloromethane, and the aqueous phases were analyzed by HPLC-MS to detect compounds **5–7**.

### Feeding experiments with detached leaves

Leaves from six to eight-week-old *A. thaliana* Col-0, 35S:CYP79A2, 35S:CYP79A2×*Nit2*-RNAi (F1), *T. majus*, or *N. officinale* were fed to late instar *P. rapae* larvae that had been starved overnight. A defined amount of leaf material (150–200 mg) was fed to the larvae and feces were collected until the gut was empty. Feces were resuspended in 1 ml water, solutions of ^13^C_2_-labeled standards were added to the final concentrations given below as well as 50 µl benzonitrile (1∶10,000 (vol∶vol) dilution in methanol), and the samples were extracted with dichloromethane/water. Aqueous phases were centrifuged and analyzed by GC-MS and HPLC-MS. Final concentrations of ^13^C-labeled standards were as follows: 20 µM *N*-benzoyl-^13^C_2_-glycine, and 0.13 µM *N*-^13^C_2_-phenylacetylglycine (Col-0); 100 µM *N*-benzoyl-^13^C_2_-glycine, and 8.3 µM *N*-^13^C_2_-phenylacetylglycine (35S:CYP79A2); 200 µM *N*-benzoyl-^13^C_2_-glycine, and 33.3 µM *N*-^13^C_2_-phenylacetylglycine (35S:CYP79A2×*Nit2*-RNAi (F1)); 300 µM *N*-benzoyl-^13^C_2_-glycine, and 33.3 µM *N*-^13^C_2_-phenylacetylglycine (*T. majus*); 26 µM *N*-benzoyl-^13^C_2_-glycine, 190 µM *N*-^13^C_2_-phenylacetylglycine, and 105 µM and *N*-(3-phenylpropionyl)- ^13^C_2_-glycine (*N. officinale*).

### Isolation of microsomes

Microsomes were prepared as described in [52]. Midguts were removed from larvae (fourth-fifth instar) and the contents were rinsed out with 0.5 mM DTT, 1 mM EDTA in 50 mM potassium phosphate buffer (pH 7.8). Homogenization was carried out on ice with a teflon tipped Potter-Elvehjem homogenizer in 0.5 mM DTT, 1 mM EDTA in 50 mM potassium phosphate buffer (pH 7.8) to which a few grains of PMSF had been added. To remove cellular debris, the extract was centrifuged at 3,000×g for 15 min at 4°C. The supernatant was centrifuged at 10,000×g for 30 min at 4°C. The supernatant obtained was centrifuged at 100,000×g for 1 hour at 4°C. The resulting microsomal pellet was resuspended in 50 mM potassium phosphate buffer (pH 7.8) containing 30% (vol/vol) glycerol, 200 mM sucrose, 0.5 mM DTT, 1 mM EDTA and a few crystals of PMSF using a fine haired paint brush and a Potter-Elvehjem homogenizer.

### CytP450 assays

Assays were performed in 50 mM KP_i_ buffer (pH 7.8) supplemented with 0.5 mM DTT, 1 mM EDTA, 30% (vol/vol) glycerol and a few crystals of PMSF using 200 µl microsomes in a total volume of 250 µl. As substrates, 0.5 mM phenylacetonitrile or 0.5 mM 3-phenylpropionitrile and 1.5 mM NADPH were added. Phenylacetonitrile and 3-phenylpropionitrile were dissolved in methanol to 1% (vol/vol). An appropriate amount of this stock was added to the assay mixture. As negative controls, assay mixtures containing boiled microsomes (95°C for 5 min) or no NADPH were included. To test CO inhibition, assay mixtures were flushed with CO for 1 min prior to addition of substrates. After incubation at 31°C for 45 min, 50 µl benzonitrile standard (1∶10,000 (vol∶vol) dilution in methanol) were added, and the samples were extracted twice with dichloromethane. The organic phase was dried over Na_2_SO_4_ and analyzed by GC-MS.

### Cyanide detection

The method was adapted from [30]. CytP450 assays were performed as described above, stopped with 250 µl methanol followed by the addition of 160 µl 4 mM 2,3-naphthalenedicarboaldehyde and 160 µl 5 mM glycine (^13^C_2_ or ^12^C_2_). Supernatants obtained after centrifugation were analyzed by HPLC-MS.

### Insect performance tests

First instar larvae of *P. rapae* or *S. littoralis* were allowed to feed on either *A. thaliana* wildtype or 3x/dhurrin plants (six to eight-week-old) *ad libitum*. After ten days, surviving larvae were counted and weighted.

### Feeding experiments with intact plants for detection of β-cyanoalanine

Freshly hatched *P. rapae* larvae were transferred to six to eight-week-old *A. thaliana* Col-0, 35S:CYP79A2 or 3x/dhurrin plants. After nine to ten days of feeding, larvae were frozen in liquid nitrogen, ground, suspended in dichloromethane and extracted twice with water. The aqueous phases were centrifuged and the supernatants analyzed by HPLC-MS.

### Fumigation experiments with [^15^N]HCN

A gauze-net covered beaker containing four fourth instar *P. rapae* larvae was placed inside a canning jar. HCN was released by adding 5 µl concentrated sulfuric acid to a glass vial containing 50 µl of 10 mg/ml aqueous [^15^N]KCN inside the canning jar. After 24 h, larvae were removed, frozen in liquid nitrogen and kept at −80°C before suspension in dichloromethane and double extraction with water. The aqueous extracts were analyzed by HPLC-MS.

## Supporting Information

Figure S1 **Qualitative analysis of aromatic glucosinolate metabolites in** ***P. rapae*** **feces extracts.** Feces samples were from larvae that had fed Col-0 leaves to which 2-phenylethylglucosinolate **2** (**A**), or benzylglucosinolate **1** (**B**) had been applied, or from larvae that had ingested leaves with no added glucosinolate (**C**). Glycine conjugates **5–7** (Fig. 1C) were detected by HPLC-MS. Shown are MRM traces that do not reflect quantitative ratios of **5–7** due to different MS responses and external application of glucosinolates.
(TIF)

Figure S2 **The** ***A. thaliana*** **35S:CYP79A2×Nit2-RNAi double mutant produces benzylglucosinolate, but lacks nitrilase activity.** **A.** Freeze-dried leaves of F1 plants from a cross of *A. thaliana* 35S:CYP79A2 and *A. thaliana* Nit2-RNAi were extracted in methanol. Shown are representative LC-MS MRM traces obtained with these extracts in comparison to traces obtained from extracts of *A. thaliana* 35S:CYP79A2 and wildtype Col-0. The solid line represents benzylglucosinolate **1**, while the dashed line represents the internal standard (4-hydroxybenzylglucosinolate). On average, 35S:CYP79A2 plants contained 2.1±1.0 nmol/mg f.w. benzylglucosinolate while the F1 plants (35S:CYP79A2×Nit2-RNAi) contained 1.1±0.5 nmol/mg f.w. as determined by HPLC-DAD of the desulfoglucosinolates [53]. **B.** Nitrilase activity was measured in leaf homogenates using 2.5 mM 3-phenylpropionitrile **4** as the substrate. Assays were incubated for 45 min at 37°C and 2-methoxybenzoic acid was added as an internal standard after termination of the assay with dichloromethane. The organic phases were dried over Na_2_SO_4_, derivatized with diazomethane and subsequently analyzed by GC-MS. Shown are representative GC-MS-traces (total ion current). The product of the nitrilase-catalyzed reaction, 3-phenylpropionic acid **14**, was identified as a methylated product in assay mixtures with 35S:CYP79A2 leaf homogenates but not from assay mixtures with 35S:CYP79A2×Nit2-RNAi or Nit2-RNAi leaf homogenates.
(TIF)

Table S1 **Growth of** ***Pieris rapae*** **and** ***S. littoralis*** **on wildtype and transgenic dhurrin-producing** ***A. thaliana*** **plants.** First instar larvae of *P. rapae* (experiments 1–3) or *S. littoralis* (experiments 4–6) were allowed to feed on plants of one of the two genotypes of *A. thaliana ad libitum*. After 10 d, surviving larvae were counted and weighted. The number of individuals at the beginning of each experiment is listed in the third column. Larval weight at day 10 is given as means ± SEM for each experiment and plant genotype. N is the number of data points (number of surviving individuals). Larval weight was tested for significant differences using t-Test (experminent 1, normally distributed) and Mann-Whitney U-Test (experiments 2–6).
(DOC)
