# Supplementary figures and images for: Turning the ‘Mustard Oil Bomb’ into a ‘Cyanide Bomb’: Aromatic Glucosinolate Metabolism in a Specialist Insect Herbivore
Source: PLoS One. 2012 Apr 20;7(4):e35545. doi: 10.1371/journal.pone.0035545 (PMC3334988; doi:10.1371/journal.pone.0035545)

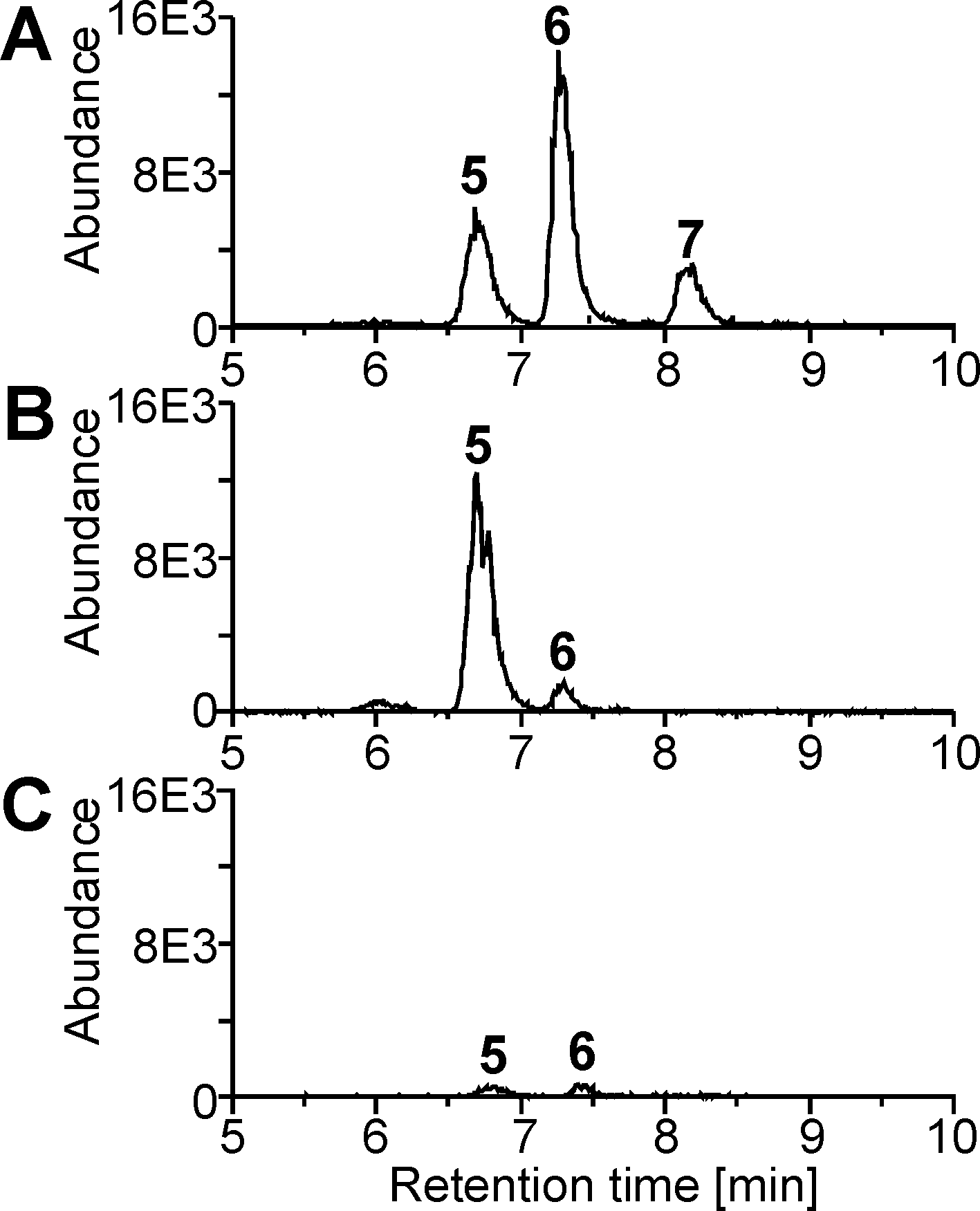

Supplement: Figure S1 — Qualitative analysis of aromatic glucosinolate metabolites in P. rapae feces extracts. Feces samples were from larvae that had fed Col-0 leaves to which 2-phenylethylglucosinolate 2 (A), or benzylglucosinolate 1 (B) had been applied, or from larvae that had ingested leaves with no added glucosinolate (C). Glycine conjugates 5–7 (Fig. 1C) were detected by HPLC-MS. Shown are MRM traces that do not reflect quantitative ratios of 5–7 due to different MS responses and external application of glucosinolates. (TIF) [file pone.0035545.s001.tif]

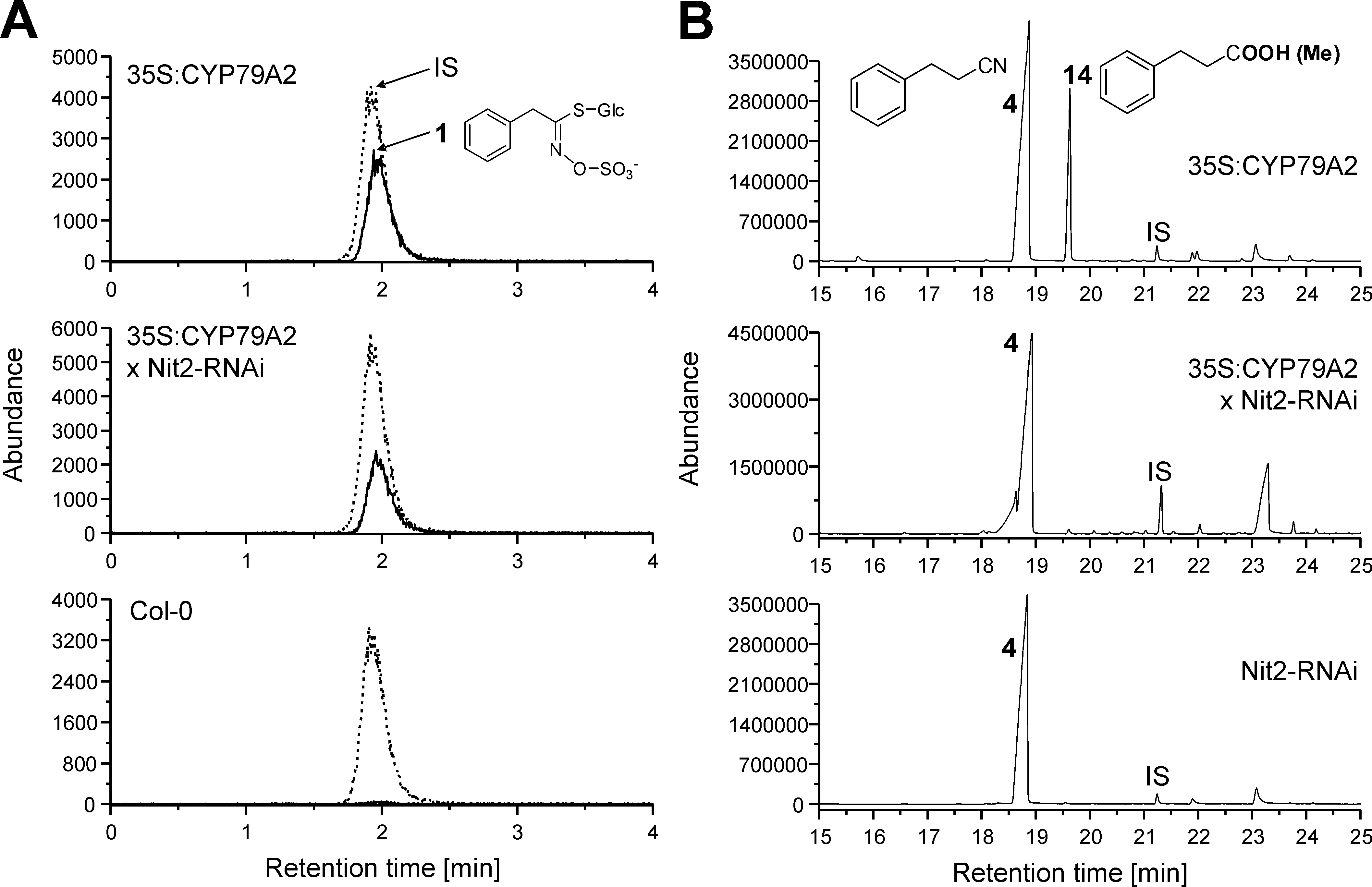

Supplement: Figure S2 — The A. thaliana 35S:CYP79A2×Nit2-RNAi double mutant produces benzylglucosinolate, but lacks nitrilase activity. A. Freeze-dried leaves of F1 plants from a cross of A. thaliana 35S:CYP79A2 and A. thaliana Nit2-RNAi were extracted in methanol. Shown are representative LC-MS MRM traces obtained with these extracts in comparison to traces obtained from extracts of A. thaliana 35S:CYP79A2 and wildtype Col-0. The solid line represents benzylglucosinolate 1, while the dashed line represents the internal standard (4-hydroxybenzylglucosinolate). On average, 35S:CYP79A2 plants contained 2.1±1.0 nmol/mg f.w. benzylglucosinolate while the F1 plants (35S:CYP79A2×Nit2-RNAi) contained 1.1±0.5 nmol/mg f.w. as determined by HPLC-DAD of the desulfoglucosinolates [53]. B. Nitrilase activity was measured in leaf homogenates using 2.5 mM 3-phenylpropionitrile 4 as the substrate. Assays were incubated for 45 min at 37°C and 2-methoxybenzoic acid was added as an internal standard after termination of the assay with dichloromethane. The organic phases were dried over Na2SO4, derivatized with diazomethane and subsequently analyzed by GC-MS. Shown are representative GC-MS-traces (total ion current). The product of the nitrilase-catalyzed reaction, 3-phenylpropionic acid 14, was identified as a methylated product in assay mixtures with 35S:CYP79A2 leaf homogenates but not from assay mixtures with 35S:CYP79A2×Nit2-RNAi or Nit2-RNAi leaf homogenates. (TIF) [file pone.0035545.s002.tif]
