# Supplementary material for: Turning the ‘Mustard Oil Bomb’ into a ‘Cyanide Bomb’: Aromatic Glucosinolate Metabolism in a Specialist Insect Herbivore
Source: PLoS One. 2012 Apr 20;7(4):e35545. doi: 10.1371/journal.pone.0035545 (PMC3334988; doi:10.1371/journal.pone.0035545)
Supplement: Table S1 — Growth of Pieris rapae and S. littoralis on wildtype and transgenic dhurrin-producing A. thaliana plants. First instar larvae of P. rapae (experiments 1–3) or S. littoralis (experiments 4–6) were allowed to feed on plants of one of the two genotypes of A. thaliana ad libitum. After 10 d, surviving larvae were counted and weighted. The number of individuals at the beginning of each experiment is listed in the third column. Larval weight at day 10 is given as means ± SEM for each experiment and plant genotype. N is the number of data points (number of surviving individuals). Larval weight was tested for significant differences using t-Test (experminent 1, normally distributed) and Mann-Whitney U-Test (experiments 2–6). (DOC) [file pone.0035545.s003.doc]

**Table S1: Growth of *Pieris rapae* and *S. littoralis* on wildtype and transgenic dhurrin-producing *A. thaliana* plants.** First instar larvae of *P. rapae* (experiments 1-3) or *S. littoralis* (experiments 4-6) were allowed to feed on plants of one of the two genotypes of *A. thaliana* *ad libitum*. After 10 d, surviving larvae were counted and weighted. The number of individuals at the beginning of each experiment is listed in the third column. Larval weight at day 10 is given as means ± SEM for each experiment and plant genotype. N is the number of data points (number of surviving individuals). Larval weight was tested for significant differences using t-Test (experminent 1, normally distributed) and Mann-Whitney U-Test (experiments 2-6).

| Experiment | Species | Number of individuals | Larval weight on Col-0  mean±SEM (N) | Larval weight on 3x/dhurrin-plants  mean±SEM (N) | p |
| --- | --- | --- | --- | --- | --- |
| 1 | *P. rapae* | 20 | 0.147±0.009 (19) | 0.138±0.011 (18) | 0.548 |
| 2 | *P. rapae* | 40 | 0.149±0.005 (40) | 0.163±0.004 (39) | 0.008 |
| 3 | *P. rapae* | 36 | 0.161±0.008 (25) | 0.155±0.010 (29) | 0.903 |
| 4 | *S. littoralis* | 33 | 0.119±0.012 (30) | 0.065±0.010 (19) | 0.004 |
| 5 | *S. littoralis* | 20 | 0.094±0.014 (20) | 0.057±0.010 (16) | 0.052 |
| 6 | *S. littoralis* | 60 | 0.033±0.003 (51) | 0.017±0.003 (45) | <0.001 |
